# Identifying Informal Help‐Seeking Patterns in African American Couples

**DOI:** 10.1111/jmft.70008

**Published:** 2025-03-11

**Authors:** Aimee Hubbard, Steven Harris, Chalandra M. Bryant, Rachel Rineman, Doneila McIntosh

**Affiliations:** ^1^ Kansas City Relationship Institute; ^2^ Department of Family Social Science University of Minnesota Saint Paul MN USA

**Keywords:** African American, couple help‐seeking, discrimination, informal help‐seeking, racial identity, religiosity, religious help‐seeking

## Abstract

While African American couples are less likely to seek formal resources, such as couples therapy, that does not mean they do not seek relationship support. The literature suggests that informal or community resources play a large role in supporting African American couples. Yet, up to this point, quantitative research has yet to identify specific factors that increase informal couple help‐seeking for African Americans. To address this gap, we examine how discrimination, racial identity, and religiosity are associated with informal couple help‐seeking. We use two distinct types of common informal couple help‐seeking (1) seeking help from a religious community and (2) seeking help from family and friends. Our study also attends to a meaningful aspect of couple help‐seeking—relationship interdependence—via an actor‐partner interdependence model (APIM). Our findings highlight the importance of informal resources in supporting African American relationships and the interdependent nature of couple help‐seeking.

Romantic relationships play a significant role in supporting individuals, as evidenced by existing research indicating that couples therapy improves individuals' depression (Beach and Whisman 2012; Carr 2019). Supporting romantic relationships is important, and its importance might be even greater for African American couples facing race‐related stressors, which can negatively impact their relationships. Research suggests that African American couples might be unaware of the detrimental effects race‐related stress can have on their physical, mental, and relational health (Hardy and Awosan 2019). Consequently, exploring ways of alleviating the negative impacts of racial discrimination for African American couples is essential. For instance, support from formal services, such as mental health providers, can mitigate the negative consequences of discrimination on individual mental health (McNeil Smith et al. 2020; Kaslow et al. 2023). However, African American couples face unique barriers in seeking formal support services (i.e., couples therapy) for their relationships, and therefore, often rely on informal networks, such as family, friends, and clergy (Hubbard et al. 2024; Vaterlaus et al. 2015). Unfortunately, factors influencing the preference for different types of informal support over formal support among African American couples remain largely understudied.

While there are a variety of ways to seek informal support for a relationship, this study specifically examines two informal resources that African American couples utilize—family/friends and religious resources (Vaterlaus et al. 2015; Hubbard et al. 2024). Earlier work indicated that African American couples are more likely to seek religious resources than therapy to support their relationships (Allen et al. 2010; Vaterlaus et al. 2015). While higher rates of religiosity among African Americans could contribute to a higher level of religious help‐seeking, there is also a cultural tendency in African American communities to favor friends and family in support of their relationship (Yeager and Williams 2022). Up to this point, researchers have not been able to identify factors that drive African Americans to intentionally seek out family/friends compared to religious resources. This study will address this important gap in the literature, as it will be the first to identify factors that drive specific types of informal couple help‐seeking behaviors among African American couples.

Race is an important factor to consider in African American help‐seeking behaviors. African Americans are less likely than other racial/ethnic groups to seek formal help (Woodward et al. 2010). Hence, race‐related factors are likely associated with African Americans choosing to rely more heavily on kinship resources (i.e., friends/family) instead of mental health professionals. While research demonstrates that discrimination negatively impacts African American individuals (Williams and Mohammed 2009) and couples (Lavner et al. 2018), less is known about whether experiences with discrimination discourage formal couple help‐seeking or if there are other race‐related factors driving these behaviors.

Race‐related factors, such as racial identity and discrimination, may impact African American couple help‐seeking (AACHS). Racial identity can act as a protective factor, creating a positive self‐concept, community, and can help individuals cope with racism (Hopkins and Shook 2017). It can also be a mechanism for increasing African American engagement with formal resources (i.e., therapist) when Black counselors are available (Townes et al. 2009); with the findings suggesting individuals with strong racial identities might feel counselors who share their racial background are more likely to understand their cultural experiences and provide relevant support (Townes et al. 2009). That lack of understanding also relates to discrimination as a possible factor in the help‐seeking process. African Americans report concerns about not being understood or discriminated against as a barrier to seeking out formal services (Hubbard et al. 2024; Vaterlaus et al. 2015). Race clearly influences the help‐seeking process, but it has yet to be explicitly explored in the AACHS process. This study will address this gap by addressing both racial identity and discrimination.

Another commonly overlooked factor in understanding the couple's help‐seeking process at large, and AAHCA, is interdependence. Interdependence in a relationship means looking at how the traits and behaviors of each person impact how the other behaves. For example, women's higher empathy is linked to men's relationship quality (Ulloa et al. 2017), and men's relationship satisfaction increases women's sexual satisfaction (Vowels and Mark 2020). Yet, less is known about how partners impact one another in the couple's help‐seeking process. To fully understand AACHS it is important to attend to the role of interdependence. This is challenging though, due to the complexity and large sample sizes required for the advanced analyses needed. Our study was able to attend to this gap as we utilized an actor‐partner interdependence model (APIM), allowing us to investigate the interdependent relationship of the predictors outlined above with two types of informal couple help‐seeking. The use of an APIM analysis is unique and will help to demonstrate the interdependent nature of AACHS.

## 1 Literature Review

### 1.1 African American Informal Help‐Seeking

Despite the need for support, African American couples demonstrate low participation in couples therapy (Dixon 2009; Vaterlaus et al. 2015). Instead, African Americans predominantly rely on informal support networks, including family, friends, and church‐based social networks (Allen et al. 2010; McElroy‐Heltzel et al. 2018; Mussa et al. 2023). This inclination toward informal sources of support is undoubtedly influenced by cultural norms promoting strong kinship bonds and filial responsibility within the African American community (Hays and Lincoln 2017). Strong kinship ties, as exemplified by extended family and church‐based networks, are vital resources for African Americans, offering an array of support, including instrumental (e.g., child‐care), emotional, social, and psychological assistance and resources (Chatters et al. 2002; Nguyen et al. 2016). Chatters et al. (2002) found that 55.3% of African Americans received informal support from both church‐based networks and family members, 27% from family members alone, and 8% from church members alone. These findings all support the strong influence of both religious and relational (i.e., friends and family) supports in the lives of African Americans.

The limited literature on AACHS shows that African American couples seek out these informal resources for relationship support in lieu of couples therapy. A seminal study by Vaterlaus et al. (2015) found that African American couples seldom turn to couples therapy (CT) for relationship support, primarily citing concerns about the trustworthiness and credibility of couple therapists. Notably, disengagement from couples therapy does not suggest an absence of help‐seeking behaviors; rather, these couples sought support from alternative resources within their social networks, relying on relational help‐seeking (i.e., friends/family) as well as religious sources (Vaterlaus et al. 2015).

The literature addressing AACHS clearly highlights the importance of religion and religious support. African Americans demonstrate significantly higher levels of religiosity compared to the general population, with a vast majority citing religion as profoundly significant in their lives (Pew Research Center 2016). African Americans also have higher levels of religious participation (church attendance, frequency of prayer, and self‐assessed religiosity) than the general population (Hays and Lincoln 2017; Moore et al. 2021). Religion holds a pivotal role in the lives of many African Americans, as evidenced by Phillips et al.'s (2012) study of 142 married African American couples with enduring marriages (i.e., 15‐60 years). In this study, 51% of the couples surveyed credited their deep belief in religion as the primary factor that sustained their marriages (Phillips et al. 2012). Consequently, it is unsurprising that pastors and church leaders are a significant and consistent source of support among African American couples (Vaterlaus et al. 2015). Clergy members are often trusted figures within African American communities (Allen et al. 2010) and are more readily accessible than mental health providers (Dempsey et al. 2016). Additionally, clergy members are culturally accepted as reliable sources of guidance for navigating relational difficulties (Wilmoth and Blaney 2016). Given all of this, the prevalence of religious couple help‐seeking by African American couples is not surprising. To better understand religious couple help‐seeking this study specifically looks at seeking help from religious leaders or someone else in the religious community.

Seeking help from religious leaders or a religious community is also distinct from relational help‐seeking (i.e., family and friends). A recent qualitative study included a sample of African Americans that identified as not religious or mildly religious, and these participants reported seeking family and friends as a significant source of support (Hubbard et al. 2024). Even in a sample that is highly religious, couples frequently reported on the importance of seeking out family and friends during moments of relationship distress (Vaterlaus et al. 2015). Together, these findings demonstrate the important role of family and friends in supporting African American romantic relationships, and that it is conceptually distinct from religious help‐seeking.

### 1.2 Discrimination

Racial discrimination remains a frequent and ubiquitous force among African Americans (Lavner et al. 2018). Scholars assert that negative racial experiences, such as discrimination, accumulate over time and create a distinct form of stress (e.g., race‐based stress) that renders African Americans, including African American couples, uniquely vulnerable (Awosan and Opara 2016; Awosan and Hardy 2017; Nightingale et al. 2019). The negative sequelae of chronic exposure to racial discrimination on physical and mental health have been well documented in extant literature (Carter et al. 2017). Moreover, exposure to discrimination is associated with increased stress which compounds otherwise typical relational stressors (Berger and Sarnyai 2015; Nightingale et al. 2019). Suffice it to say discrimination is a critical variable to attend to when trying to understand African Americans' lived experiences.

Due to the ubiquitousness of racial discrimination and racial stress, African American couples may be unaware of the toll that race‐related stress has on their relationships, attributing their negative interaction patterns to their partner's shortcomings (Hardy and Awosan 2019). This can lead to relational distress for Black women and foster anger in Black men (Awosan et al. 2011; Kogan et al. 2016; Woods‐Giscombé 2010). Concerns related to racism are barriers to seeking couples therapy, due to the absence of culture/racial matching with clinicians (Hubbard et al. 2024). African American couples report wanting to work with a clinician who shares their lived experience of race, a reality that may not be accessible to many couples (Hubbard et al. 2024; Vaterlaus et al. 2015). Therefore, it is important to note that due to experiences of discrimination, African American couples may seek out informal support due to concerns that they may experience discrimination or receive formal support from mental health providers who are not versed in addressing race‐related distress.

### 1.3 Racial Identity

Racial identity is a person's sense of belonging to a particular racial group (Phinney 1990), and past research indicates that racial group membership has vital implications for African Americans' physical and psychological well‐being (Yap et al. 2011). For example, research suggests that African Americans' positive feelings about their racial group are consistently associated with various positive psychological outcomes, including less stress, depression, psychological distress, and higher levels of self‐esteem (Bynum et al. 2008; Settles et al. 2010; Yap et al. 2011). Moreover, racial identity is associated with help‐seeking behavior, both formal and informal (Powell et al. 2016; Richman et al. 2007). Individuals with higher levels of racial identity (e.g., strongly identify with their racial group) are likely to seek out group members through individual relationships and organizational involvement (Yap et al. 2011). Additionally, African Americans with greater racial identity also utilize family members, close friends, or trusted community members as the primary resources of assistance and help‐seeking (Powell et al. 2016; Wallace and Constantine 2005). Notably, however, these proximate resources (e.g., familial, social, religious) are often accessed and exhausted before turning to formal mental health services (Constantine et al. 2004). To the best of our knowledge, no previous research has explored how racial identity relates to African American couples' informal help‐seeking. Given the variety of findings in the broader literature and the lack of research related to couples specifically, this study seeks to clarify the role of racial identity in AACHS.

### 1.4 Theory

According to interdependence theory (Van Lange 2012), individuals' outcomes are shaped by their experiences and interactions with their partners. Individuals in committed relationships have frequent interactions with each other and are undoubtedly influenced by the thoughts, feelings, and behaviors of their partners (McNeil Smith et al. 2020). Marital relationships, therefore, have significant impacts on individuals' health, mental health (Carr 2019), as well as help‐seeking behaviors (Doss et al. 2003). Therefore, the current study seeks to examine the interdependent factors that influence how African American couples engage with religious couple help‐seeking as well as relational couple help‐seeking, here identified as friends and family support systems.

### 1.5 Current Study

The present study aims to examine how discrimination, internalized racial identity, pre‐encounter racial identity, and religiosity influence the help‐seeking behaviors of African American couples. This study focuses specifically on informal help‐seeking, particularly from family/friends and religious resources. We aim to test the following hypotheses in this analysis.

1. Discrimination will be positively associated with seeking relational support (i.e., friends and family).
2. Internalized and pre‐encounter racial identity will be positively associated with seeking religious resources.
3. Greater religiosity will be associated with seeking out religious resources.

This study seeks to address gaps in the existing literature and provide a more nuanced understanding of help‐seeking behaviors within the African American community, in particular, their use of informal support networks (i.e., religious and relational).

## 2 Methods

### 2.1 Longitudinal APIM

We used a longitudinal actor‐partner interdependence model (APIM) for this analysis. APIM uses the couple as the unit of analysis (Kenny et al. 2006), and we used sex to distinguish each member of the couple. Partner paths were included for all variables as part of the APIM analysis (Kenny et al. 2006). Unstandardized path coefficients were used, as this allows for comparisons across the husbands/wives (Kenny et al. 2006). Due to missing predictor values, there were 328 observations out of a possible 350. All correlations and descriptive data are available in supplemental tables.

### 2.2 Method

#### 2.2.1 Sample

Data were from a project funded by the National Institute of Child Health and Human Development titled *A Study of African American Marriage and Health [R01 HD050045 Eunice Kennedy Shriver National Institute of Child Health and Human Development]*. The data set has three waves of dyadic data. The sample consists of newly married heterosexual couples, married a year or less at wave one. Wave one was collected in 2005, wave two was collected approximately one year later, and wave three was collected two and one‐half years after the first wave.

### 2.3 Measures

#### 2.3.1 Racial Identity

This measure was adapted from the Black Racial Identity Scale (RIAS‐B; Helms 1990). The measure has two subscales: *Pre‐Encounter and Internalization*. The Pre‐Encounter scale included seven items, which assessed how individuals devalue their identity (e.g., “African Americans do not speak as well as Whites”). The Internalization subscale, which assessed how comfortable individuals are with their racial identity, also included seven items (e.g., “Being African American is an important part of my self‐image”). Items were coded using a 5‐point Likert scale such that higher scores reflected greater endorsement. The reliability for the Pre‐Encounter subscale at Time 1 was acceptable; Chronbach's *α* for husbands was 0.53, and for wives, 0.52. The reliability for the Internalization subscale at Time 1 was also acceptable; the Cronbach's *α* for husbands was 0.67 for husbands and 0.65 for wives.

#### 2.3.2 Discrimination

To assess for discrimination the research project used 10 items from an established measure (Murry et al. 2001). The reliability for this measure was good for both husbands (*α* = 0.83) and wives (*α* = 0.81) at Time 1. The measure assessed participants' experiences with various situations during the past year (e.g., “How often has a store owner, salesclerk, or person working at a place of business treated you in a disrespectful manner just because you are African American?”). Items were rated using a 5‐point Likert scale; higher scores reflected more experiences with discrimination.

#### 2.3.3 Religiosity

Husbands and wives responded to three continuous items that assess organizational religiosity—that is, “how often do you usually attend religious services?” Items were scored on a 4‐point Likert scale, (1 = *Always* and 4 = *Never*) Scores were reverse coded, such that higher scores reflected a higher level of religiosity. The reliability for the organizational religiosity was good for husbands (*α* = 0.74) and wives (*α* = 0.73) at time 1. To try and account for other aspects of religiosity, we included the single item, “How religious would you say you are?” A higher rating was coded to indicate a higher level of religiosity.

#### 2.3.4 Attitudes Toward Help‐Seeking From Religious Officials

Attitudes Toward Help‐Seeking From Religious Officials included two items, one aimed at intent to seek religious resources and the other assessing perceived helpfulness of religious resources. The two items were: “If I ever had marital problems, I would talk to my pastor, priest, or spiritual leader about it” and “If my marriage is ever in trouble, I know that my pastor, priest, or spiritual leader could help me save it.” Each item was assessed using a 5‐point Likert scale (1 = *Strongly Agree* and 5 = *Strongly Disagree*). Items were reverse coded; higher scores reflected a greater likelihood of eliciting the help of religious officials. The Cronbach's *α* for husbands was 0.81, and for wives, 0.77 at Time 2.

#### 2.3.5 Help‐Seeking Behaviors

Next, we measured actual help‐seeking behavior. This measure asked couples about help‐seeking behavior from various sources. Participants were asked, “have you received or are you receiving marriage counseling from…” and had six help‐seeking options. Two items assessed formal help‐seeking (seminar/workshop, psychologist/counselor/marriage therapist), two items assessed relational help‐seeking (parents/family member, friend), and two items assessed religious help‐seeking (pastor/minister/priest/rabbi, someone else in religious community). Items were dichotomous (“Yes” or “No”). Items were summed and then re‐coded so they would remain dichotomous—as the summed scores were highly skewed and kurtotic. At Time 3, 6% of husbands engaged in formal help‐seeking, 16% of husbands engaged in relational help‐seeking, and 36% of husbands engaged in religious help‐seeking. For wives at time 3, 7% engaged in formal help‐seeking, 6% of wives engaged in relational help‐seeking, and 33% of wives engaged in religious help‐seeking. Low levels of help‐seeking are consistent with extant literature.

#### 2.3.6 Covariates

Four covariates were included in the analyses: global health, education, relationship dissatisfaction, and income. These variables were selected as previous research has connected them with couple help‐seeking behavior (Doss et al. 2009; Hubbard and Harris 2020), or overall mental/physical healthcare help‐seeking (Hepworth and Paxton 2007).

##### 2.3.6.1 Global Health

Global health was assessed using three items. “How would you rate your overall physical health?”, “How would you rate your overall mental health?”, and “Would you say your overall physical health is better or worse than other people your age?” All items were coded such that higher scores reflected poor overall health. The Cronbach's alphas for wives and husbands were *α* = 0.96) and *α* = 0.65, respectively, at Time 1.

##### 2.3.6.2 Education

This is a single‐item question: “What is the highest level of education you have completed?” Participants had 10 response options ranging from “grade school” to “medical doctor/M.D.”. Responses were coded so that higher scores reflected greater levels of education.

##### 2.3.6.3 Relationship Distress

This is a single‐item question. Single‐item assessments are a valid assessment tool for relationship satisfaction (Fülöp et al. 2020). The item asked: “All things considered, how happy are you with your marriage so far?” Participants' responses to the question were rated using a 5‐point Likert‐type scale (1 = *Very Happy* and 5 = *Very Unhappy*). Responses were coded such that higher scores equated to greater relationship distress.

##### 2.3.6.4 Income

Husbands and wives reported their income rather than household income; thus, both husbands' and wives' self‐reported incomes were used as covariates for analyses.

### 2.4 Preliminary Analyses

The bivariate correlation results (see Tables 4 and 5 in supplemental materials) revealed significant associations between the variables in the proposed model. We deemed it appropriate to move forward with additional analyses based on correlations. While there was no systematic missingness, the missing data made it so the model could not converge, even with the use of auxiliary variables. As a result, we only used the data of participants who completed all three waves (*N* = 363) and had data for the outcome variables (*N* = 350). Covariates were included in the analysis to address factors that may have driven attrition. Descriptive statistics for all predictors and covariates are listed in Supporting Information S1: Table 3.

#### 2.4.1 Confirmatory Factor Analysis (CFA)

To assess the factor structure of predictor variables (religiosity, discrimination, and racial identity), we ran a CFA. The measures and sub‐scales used in this study had good model fit (Hu and Bentler 1999). One item from the discrimination variable (“How often has anyone discouraged you from trying to achieve an important goal just because you are African American?”) did not load well and negatively impacted fit. After removing the item, the model fit improved significantly, shifting from adequate to good model fit (Hu and Bentler 1999).

## 3 Results

**Figure 1:**
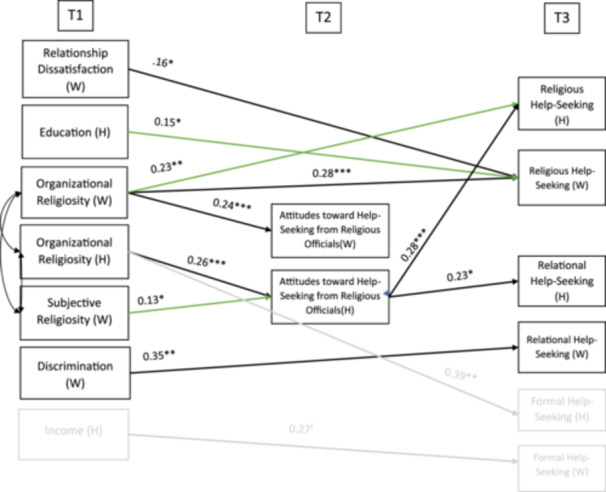
Final longitudinal APIM model for African American help‐seeking (*N* = 328). [Color figure can be viewed at wileyonlinelibrary.com]

Results of the longitudinal APIM are shown in Figure 1. Due to the complexity of this model, this study focuses only on the paths related to religious and relational help‐seeking. The paths related to formal help‐seeking are in a lighter gray in the figure. Only significant or approaching significant paths are shown in the figure. Actor paths are black, while partner paths are green. Given that the outcome variables were dichotomous, we ran a logistic model that showed the probabilities that an outcome increases because of the predictors via log‐odds ratios. Log‐odd ratios show that an increase of one in the independent variables increases the odds of the outcome at a constant rate (Kline 2011). In the logistic analysis, the model fit is determined by the loglikelihood and the Bayesian Information Criterion (BIC). Better fit is shown by lower loglikelihood and BIC values (Hilbe 2011). We systematically dropped nonsignificant paths until the BIC and loglikelihood stopped decreasing. The model with the lowest loglikelihood and BIC was selected as the final model. The final model accounted for 13.3% of wives' and 14.9% of husbands' religious help‐seeking and 8.2% of husbands' attitudes toward help‐seeking from religious officials.

### 3.1 Actor Effects

Actor effects are the direct effects of the individual's independent variables on their own dependent variable. Husband actor effects showed that their greater endorsement of organizational religiosity at Time 1 was positively associated with their attitude toward help‐seeking from a religious official at Time 2 (*β* = 0.20, *p* < 0.001). Husbands' attitude toward help‐seeking from religious officials at Time 2 was positively linked to their relational (*β* = 0.20, *p* < 0.05) and religious help‐seeking at Time 3 (*β* = 0.29, *p* < 0.01). Wives' organizational religiosity at Time 1 was also positively associated with their own attitudes toward help‐seeking from religious officials at Time 2 (*β* = 0.17, *p* < 0.001) and their own religious help‐seeking at Time 3 (*β* = 0.20, *p* < 0.001). Wives' actor effects also showed that their relationship distress at Time 1 was linked to greater religious help‐seeking at Time 3 (*β* = 0.46, *p* < 0.05). Wives who experienced greater discrimination at Time 1 were more likely to seek relational help (*β* = 0.35, *p* < 0.01) at Time 3. Wives' attitude toward help‐seeking from religious officials at Time 2 was not associated with any help‐seeking at Time 3.

### 3.2 Partner Effects

Partner effects are the direct effects of the individual's (husband or wife) independent variables on their partner's dependent variable. This model contained three significant partner effects. Wives' greater organizational religiosity at Time 1 was associated with husbands' greater religious help‐seeking at Time 3 (*β* = 0.16, *p* < 0.01). While wives' subjective religiosity at Time 1 was positively associated with husbands' attitudes toward help‐seeking at Time 2 (*β* = 0.37, *p* < 0.05). Husbands' greater education at Time 1 was associated with wives' greater religious help‐seeking (*β* = 0.16, *p* < 0.05) at Time 3.

### 3.3 Log‐Odds

The regression coefficient for each logistic regression is converted into separate odds ratios (Kline 2011). Odds ratios estimate the difference in the odds of the outcome for a one‐point difference in the predictor while controlling for other predictors (Kline 2011). Our analysis showed significant log‐odds for actor and partner effects, results are reported in Table 1.

**Table 1:** Significant log‐odds results (standardized solution, *N* = 328) (W) = wives; (H) = husbands).

Predictors	Outcomes	Estimate	SE	*p* value
(W) Organizational Religiosity	(W) Religious Help‐Seeking	1.217	0.069	0.002
(W) Relationship Dissatisfaction	(W) Religious Help‐Seeking	1.586	0.276	0.034
(W) Education	(W) Religious Help‐Seeking	1.174	0.086	0.043
(W) Organizational Religiosity	(H) Religious Help‐Seeking	1.179	0.063	0.004
(H) Attitudes toward help‐seeking from religious officials	(H) Religious Help‐Seeking	1.289	0.085	0.001
(W) Discrimination	(W) Relational Help‐Seeking	1.169	0.063	0.007
(H) Attitudes toward help‐seeking from religious officials	(H) Relational Help‐Seeking	1.220	0.097	0.024
(H) Organizational religiosity	(H) Formal Help‐Seeking	1.336	0.149	0.024

### 3.4 Indirect Effects

We tested indirect paths with bootstrapping procedures (Preacher and Hayes 2008). Two indirect paths were significant; both were actor effects. The first was husbands' organizational religiosity →husbands' attitudes toward help‐seeking from religious officials → husbands' religious help‐seeking (*β* = 0.04, *p* < 0.05, CI 0.002.078). The second was husbands' organizational religiosity, →husbands' attitudes toward help‐seeking from religious officials husbands' religious help‐seeking (*β* = 0.05, *p* < 0.01, CI 0.015.081). Using this last pathway as an example, a one standard deviation increase in husbands' organizational religiosity is associated with a 0.05 standard deviation increase in husbands' relational help‐seeking due to the prior effect of husbands' attitudes toward help‐seeking from religious officials. All indirect effects can be seen in Table 2.

**Table 2:** Significant indirect effects (standardized solution, *N* = 328).

Predictors	Mediator(s)	Outcomes	β	CI		*p* value
(H) Organizational Religiosity →	(H) Attitudes toward Help‐seeking from Religious Officials →	(H) Relational Help‐Seeking	0.040	0.002	0.078	0.040
(H) Organizational Religiosity →	(H) Attitudes toward Help‐seeking from Religious Officials →	(H) Religious Help‐Seeking	0.051	0.015	0.081	0.005

*Note:* Indirect paths tested with 2000 bootstraps.

Abbreviations: CI = 95% confidence interval; (H), husbands; (W), wives.

## 4 Discussion

This study fills meaningful gaps in the limited research on African American couples' help‐seeking. To start, our study suggests that a race‐related factor (discrimination) was significantly associated with relational help‐seeking. Additionally, using an APIM allowed us to explore the interdependent nature of help‐seeking, highlighting how partner effects can impact African American couples' help‐seeking (AACHS). We hope future researchers will build on these findings and expand this limited body of literature.

As hypothesized, experiences with discrimination were associated with informal couple help‐seeking from friends and family. Interestingly, this effect was present only among wives. While women tend to confide in, and be confided in, more often than men (Lind Seal et al. 2016), other investigations found no gender differences among African American men and women in relational help‐seeking regarding their romantic relationships (Yeager and Doherty 2022; Woodward et al. 2010). Perhaps African American men who have experienced greater instances of discrimination feel less comfortable asking for advice about their romantic relationships in general. While masculinity has a negative association with couple help‐seeking behavior (Parnell and Hammer 2018), there is no empirical research specifically investigating how masculinity may uniquely impact relational help‐seeking among African American men. Future research should consider explicitly exploring the interaction between gender (masculinity), discrimination, and couple help‐seeking.

The role of racial identity in help‐seeking behaviors is somewhat unclear in the literature. Due to the central role of religiosity, kinship, and familial relationships in African American culture (Hays and Lincoln 2017; Moore et al. 2021), we expected those with higher internalized racial identities to seek more informal help. However, we found no significant association between pre‐encounter or internalized racial identity with any informal type of couple help‐seeking. There are some possible explanations for the lack of significant findings in the present study. When an individual model was run, husbands' pre‐internalized racial identity approached significance for religious help‐seeking. The lack of significant paths in the full model suggests weak associations. Given these results and the lower (though acceptable) reliability, it could be that the racial identity measure does not fully capture the nuances of this trait. An earlier study hypothesized that the measure represents a state versus variable trait, which may account for the lack of consistency (Lemon and Waehler 1996). Another explanation for the lack of significant findings is that while our sample was large and had sufficient power, it was not robust enough to detect the impact of racial identity. Even with these nonsignificant findings, this is the first empirical study to explore the relationship between racial identity and informal help‐seeking among married African American couples. While further research is needed to understand racial identity's role in informal couple help‐seeking, this study is an important first step.

As expected, organizational religiosity was associated with seeking help from religious resources. These findings are aligned with previous work by Hardy (2012), in which African Americans overwhelmingly stated religious leaders as the preferred resource for marriage support for most issues (e.g., general marital issues, infidelity, and abuse). Interestingly though, a direct effect of organizational religiosity was found only among wives. Women tend to be the ones to reach out for marital support in general (Doss et al. 2003). It is possible that African American women parallel this finding, but with a preference for seeking out religious resources.

Conversely, there was no direct effect of organizational religiosity on husbands' help‐seeking, but there was an indirect effect of positive attitudes toward help‐seeking from religious leaders. This means that when husbands held positive attitudes about seeking help from religious leaders, their help‐seeking from religious resources increased. This suggests that these positive attitudes are a mechanism through which African American men seek marital support from religious resources. These findings may point to an important aspect of AACHS: trust and comfort in the resource being sought out. Research has shown the importance of speaking to trusted individuals (e.g., pastors or marriage counselors) about emotional or marital struggles (Hardy 2012). Connected to trust, it is also possible that African American men who sought help from religious resources had positive experiences seeking help from religious leaders, or they personally knew someone who sought help this way. This is consistent with research showing the importance of modeling help‐seeking behavior (Hubbard et al. 2024). These gender differences in the role of religiosity and religious help‐seeking suggest possible mechanisms for engagement by building trust and positive experiences with religious leaders. Research shows that relationship‐building, religious knowledge, confidentiality, and non‐judgment are all factors that can help build trust in religious leaders (Hardy 2012).

The partner effects that emerged from our analysis can further contextualize these findings. We found that wives' organizational religiosity increased husbands' help‐seeking from religious resources. Consistent with interdependence theory, these findings show how one partner's individual beliefs can impact their partner's behavior (Rusbult and Van Lange 2003). The broader help‐seeking literature shows women tend to lead the couple's help‐seeking processes (Doss et al. 2003); yet the couple help‐seeking literature at large has not been able to fully account for the interdependent nature of couple help‐seeking (Hubbard and Harris 2020). These findings highlight the value of using APIM analysis to attend to the interdependent nature of romantic partnerships.

This interdependence was also observed with two other variables. We found that husbands' higher educational attainment was positively associated with wives' religious help‐seeking, meaning wives sought religious resources for their relationship when their husbands had a higher level of education. Generally, higher rates of education increase exposure to and acceptance of formal help‐seeking for men, including African American men (Hammer et al. 2013), and women tend to initiate formal couple help‐seeking more often (Hubbard and Harris 2020). Taken together, this shows the intersection of education, gender, alongside African Americans' preferences for religious help‐seeking (Hays and Lincoln 2017). The present findings suggest that wives with more highly educated husbands may see their husbands as more open to seeking help, thus wives may feel more comfortable seeking help from their preferred relationship support resource.

Not surprisingly, the variable most often associated with any help‐seeking behaviors is distress. For couples, relationship distress often drives formal help‐seeking behaviors (Hubbard and Harris 2020). Consistent with this, our results suggest that when African American wives felt unhappy with their marriage, they sought help from religious resources. When paired with a cultural preference for and greater access to relationship help from religious resources (Phillips et al. 2012; Vaterlaus et al. 2015), it makes sense that relationship distress would be connected to religious help‐seeking for wives. Doss et al. (2003) found that both partners perceived wives as more aware of and engaged in seeking help for relationship issues. These findings are corroborated by the current study, which suggests that wives are more driven than husbands to seek support for couple‐related distress.

Though complex, each of these findings helps to illuminate unique factors related to AACHS. The role of racial factors, such as discrimination and racial identity, cannot be ignored and bear further investigation. The higher rate of religiosity and religious help‐seeking in the AACHS process highlights a meaningful entry point clinicians can support, which will be discussed in more depth below. African American couples face additional and unique stressors as individuals and couples. Both clinicians and researchers need to consider how they can effectively support African American couples in a culturally humble manner.

### 4.1 Clinical Implications

While couples therapists work to identify how they can increase African American engagement, the findings of our study highlight the important role of informal resources. By recognizing that many African American couples turn to religious institutions or friends/family for support, therapists can better integrate these informal resources into their clinical work. Couples therapists working with African American couples should consider assessing other types of support, including religious support or support from family and friends. Understanding the nature of this support can strengthen the therapeutic alliance by aligning therapy with the couple's lived experiences and value systems. Integrating information and outside support into couples therapy might help not only build a better alliance but also help create greater change.

Therapists also need to consider how their biases toward couples therapy might limit how they assess couples' utilization of informal resources. It is easy for therapists to assume couples therapy is the “best” option, but this stance could invalidate African American couples' experiences. Taking a culturally humble approach means taking a stance that is other‐oriented, focusing on an individual's cultural background without judgment (Hook et al. 2013). In this scenario, practicing cultural humility in couples therapy is showing respect and interest in how informal resources and institutions such as the Black Church are used to support relationships. Attention to and integration of informal support are just one way of ensuring that therapists are taking a culturally humble approach in their work.

Additionally, these findings reinforce the important role of the Black church in the lives of African Americans. Black churches emerged during the time of enslavement and have continued to be a place to express cultural heritage, develop community, and organize (Allen et al. 2010). Historically and today the Black church serves a variety of community‐level functions (Billingsley and Caldwell 1991), offers a sense of community and hope (Chaney 2008), and is a source of support for romantic relationships (Vaterlaus et al. 2015). Research also shows churches are a meaningful entry point for relationship education and support (Hurt et al. 2008). Given the literature and our findings, a practical step clinicians could consider is establishing collaborations with the Black churches in their community.

Any clinician considering building relationships with Black churches needs to read about best practices (Dempsey et al. 2016) before getting started. In particular, any non‐Black clinicians looking to form partnerships should start by reflecting on their own biases, beliefs, and assumptions. This reflection is crucial given the historical and ongoing biases within the mental health system (Kelly et al. 2020; Skott‐Myhre and Skott‐Myhre 2022). The findings from our study highlight the importance of community resources, from Black Church to relational support from friends/family. It is our hope that clinicians can use the insights from this project to build relationships within the African American community, and further support their existing resources for African American couples. We believe this approach is meaningful to cultivating greater trust and engagement in couples therapy for African American couples.

These findings also highlight places where researchers might collaborate with clinicians to better support African American couples. There is an evidence‐based program that supports relational help‐seeking, Marital First Responders (Lind Seal et al. 2016), and it has been validated among African Americans (Yeager and Doherty 2022). There are likely opportunities for researchers and clinicians to partner and better support African American couples in spaces and places they are already utilizing. Not only would this approach help develop community‐based interventions that have yet to be fully realized, but it might also be a helpful steppingstone in building trust between researchers, clinicians, and African American communities.

### 4.2 Limitations

The findings presented in this study must be considered within their limitations. To start, this study is not generalizable to all African American couples. There is great heterogeneity among African Americans, and given the participants' high religiosity and geographical centrality, these findings may not be representative of African Americans who are less religious or live in different geographic areas. Additionally, the study focused on newlyweds and may not fully capture the help‐seeking dynamics of those who have been married longer. There was also significant attrition from Time 1 to Time 3. While efforts were made to address this through an attrition analysis and the inclusion of auxiliary variables, it is possible that our results may have differed with less missing data. However, it is worth highlighting that the final sample included over 300 couples, suggesting sufficient statistical power to detect significance. Lastly, while this study looked at common informal resources (friends/family and religious community), it did not encompass all informal resources (books, movies, blogs, social media, etc.). Future research should consider exploring a wider variety of informal resources. Notwithstanding these constraints, our findings offer meaningful potential avenues for future research and can inform therapists of possible mechanisms for supporting African American couples.

## 5 Conclusion

The findings from this study emphasize the importance of informal resources for African American romantic relationships. Given the limited research on African American couples' help‐seeking, this study significantly expands our knowledge of what traits drive informal help‐seeking. In particular, the focus on informal resources supports the important role of kinship in the African American community. Moreover, this study highlights the interdependent nature of couples with three significant partner effects: wives' religiosity, relationship distress, and husbands' level of education. The authors hope other researchers can build on our findings to identify ways policy, communities, and mental health providers will better support African American couples in respectful and culturally appropriate ways.

## Supporting information

Supporting information.

## References

[jmft70008-bib-0001] Allen, A. J. , M. P. Davey , and A. Davey . 2010. “Being Examples to the Flock: The Role of Church Leaders and African American Families Seeking Mental Health Care Services.” Contemporary Family Therapy 32, no. 2: 117–134. 10.1007/s10591-009-9108-4.

[jmft70008-bib-0002] Awosan, C. I. , and K. V. Hardy . 2017. “Coupling Processes and Experiences of Never Married Heterosexual Black Men and Women: A Phenomenological Study.” Journal of Marital and Family Therapy 43, no. 3: 463–481. 10.1111/jmft.12215. 28205237

[jmft70008-bib-0003] Awosan, C. I. , and I. Opara . 2016. “Socioemotional Factor: A Missing Gap in Theorizing and Studying Black Heterosexual Coupling Processes and Relationships.” Journal of Black Sexuality and Relationships 3, no. 2: 25–51. 10.1353/bsr.2016.0027.29201951

[jmft70008-bib-0004] Awosan, C. I. , J. G. Sandberg , and C. A. Hall . 2011. “Understanding the Experience of Black Clients in Marriage and Family Therapy.” Journal of Marital and Family Therapy 37, no. 2: 153–168. 10.1111/j.1752-0606.2009.00166.x. 21457281

[jmft70008-bib-0005] Beach, S. R. H. , and M. A. Whisman . 2012. “Affective Disorders.” Journal of Marital and Family Therapy 38, no. 1: 201–219. 22283387 10.1111/j.1752-0606.2011.00243.x

[jmft70008-bib-0006] Berger, M. , and Z. Sarnyai . 2015. “‘More Than Skin Deep’: Stress Neurobiology and Mental Health Consequences of Racial Discrimination.” Stress 18, no. 1: 1–10. 10.3109/10253890.2014.989204. 25407297

[jmft70008-bib-1007] Billingsley, A. , and C. H. Caldwell . 1991. “The Church, the Family, and the School in the African American community.” Journal of Negro Education 60, no. 3: 427–440.

[jmft70008-bib-0007] Bynum, M. S. , C. Best , S. L. Barnes , and E. T. Burton . 2008. “Private Regard, Identity Protection and Perceived Racism Among African American Males.” Journal of African American Studies 12: 142–155. 10.1007/s12111-008-9038-5.

[jmft70008-bib-0008] Carr, A. 2019. “Couple Therapy, Family Therapy and Systemic Interventions for Adult‐Focused Problems: The Current Evidence Base.” Journal of Family Therapy 41, no. 4: 492–536. 10.1111/1467-6427.12225.

[jmft70008-bib-0009] Carter, R. T. , M. Y. Lau , V. Johnson , and K. Kirkinis . 2017. “Racial Discrimination and Health Outcomes Among Racial/Ethnic Minorities: A Meta‐Analytic Review.” Journal of Multicultural Counseling and Development 45, no. 4: 232–259. 10.1002/jmcd.12076.

[jmft70008-bib-0010] Chaney, C. D. 2008. “The Benefits of Church Involvement for African‐Americans: The Perspectives of Congregants, Church Staff, and the Church Pastor.” Journal of Religion and Society 10: 1–23.

[jmft70008-bib-0011] Chatters, L. M. , R. J. Taylor , K. D. Lincoln , and T. Schroepfer . 2002. “Patterns of Informal Support From Family and Church Members Among African Americans.” Journal of Black Studies 33, no. 1: 66–85. 10.1177/002193470203300104.

[jmft70008-bib-0012] Constantine, M. G. , L. J. Myers , M. Kindaichi , and J. L. Moore, III . 2004. “Exploring Indigenous Mental Health Practices: The Roles of Healers and Helpers in Promoting Well‐Being in People of Color.” Counseling and Values 48, no. 2: 110–125. 10.1002/j.2161-007X.2004.tb00238.x.

[jmft70008-bib-0013] Dempsey, K. , S. K. Butler , and L. Gaither . 2016. “Black Churches and Mental Health Professionals: Can This Collaboration Work?” Journal of Black Studies 47, no. 1: 73–87. 10.1177/0021934715613588.

[jmft70008-bib-0014] Dixon, P. 2009. “Marriage Among African Americans: What Does the Research Reveal?” Journal of African American Studies 13, no. 1: 29–46. 10.1007/s12111-008-9062-5.

[jmft70008-bib-0015] Doss, B. D. , D. C. Atkins , and A. Christensen . 2003. “Who's Dragging Their Feet? Husbands and Wives Seeking Marital Therapy.” Journal of Marital and Family Therapy 29, no. 2: 165–177. 10.1111/j.1752-0606.2003.tb01198.x. 12728776

[jmft70008-bib-0016] Doss, B. D. , G. K. Rhoades , S. M. Stanley , and H. J. Markman . 2009. “Marital Therapy, Retreats, and Books: The Who, What, When, and Why of Relationship Help‐Seeking.” Journal of Marital and Family Therapy 35, no. 1: 18–29. 10.1111/j.1752-0606.2008.00093.x. 19161581

[jmft70008-bib-0017] Fülöp, F. , B. Bőthe , É. Gál , J. Y. A. Cachia , Z. Demetrovics , and G. Orosz . 2020. “A Two‐Study Validation of a Single‐Item Measure of Relationship Satisfaction: Ras‐1.” Current Psychology: 1–13. 10.1007/s12144-020-00727-y.

[jmft70008-bib-0018] Hammer, J. H. , D. L. Vogel , and S. R. Heimerdinger‐Edwards . 2013. “Men's Help Seeking: Examination of Differences Across Community Size, Education, and Income.” Psychology of Men & Masculinity 14, no. 1: 65–75. 10.1037/a0026813.

[jmft70008-bib-0019] Hardy, K. M. 2012. “Perceptions of African American Christians' Attitudes Toward Religious Help‐Seeking: Results of an Exploratory Study.” Journal of Religion & Spirituality in Social Work: Social Thought 31, no. 3: 209–225. 10.1080/15426432.2012.679838.

[jmft70008-bib-0020] Hardy, K. V. , and C. I. Awosan . 2019. “Therapy With Heterosexual Black Couples Through a Racial Lens.” In Re‐visioning Family Therapy: Race, Culture, and Gender in Clinical Practice, 2nd ed., edited by M. McGoldrick and K. V. Hardy , 419–432. Guilford Press.

[jmft70008-bib-0021] Hays, K. , and K. D. Lincoln . 2017. “Mental Health Help‐Seeking Profiles Among African Americans: Exploring the Influence of Religion.” Race and Social Problems 9, no. 2: 127–138. 10.1007/s12552-017-9193-1.

[jmft70008-bib-0022] Helms, J. E. 1990. Black and White Racial Identity: Theory, Research, and Practice. Greenwood Press.

[jmft70008-bib-0023] Hepworth, N. , and S. J. Paxton . 2007. “Pathways to Help‐Seeking in Bulimia Nervosa and Binge Eating Problems: A Concept Mapping Approach.” International Journal of Eating Disorders 40, no. 6: 493–504. 10.1002/eat.20402. 17573682

[jmft70008-bib-0024] Hilbe, J. M. 2011. “Logistic Regression.” In International Encyclopedia of Statistical Science, edited by M. Lovric , 755–758. SAGE.

[jmft70008-bib-0025] Hook, J. N. , D. E. Davis , J. Owen , E. L. Worthington, Jr. , and S. O. Utsey . 2013. “Cultural Humility: Measuring Openness to Culturally Diverse Clients.” Journal of counseling psychology 60, no. 3: 353–369. 10.1037/a0032595. 23647387

[jmft70008-bib-0026] Hopkins, P. D. , and N. J. Shook . 2017. “A Review of Sociocultural Factors That May Underlie Differences in African American and European American Anxiety.” Journal of Anxiety Disorders 49: 104–113. 10.1016/j.janxdis.2017.04.003. 28494387

[jmft70008-bib-0027] Hu, L. , and P. M. Bentler . 1999. “Cutoff Criteria for Fit Indexes in Covariance Structure Analysis: Conventional Criteria Versus New Alternatives.” Structural Equation Modeling: A Multidisciplinary Journal 6, no. 1: 1–55. 10.1080/10705519909540118.

[jmft70008-bib-0028] Hubbard, A. , and S. Harris . 2020. “A Critical Review of Help‐Seeking for Couples Therapy: Clinical Implications and Next Steps.” Contemporary Family Therapy 42, no. 2: 152–162. 10.1007/s10591-019-09521-w.

[jmft70008-bib-0029] Hubbard, A. , S. Harris , M. Dick , and D. McGee . 2024. “Understanding African American Help‐Seeking for Romantic Relationships: Advocacy, Barriers, and Considerations.” Journal of Marital and Family Therapy 50: 348–367. 10.1111/jmft.12692. 38383948

[jmft70008-bib-0030] Hurt, T. R. , K. J. Franklin , S. R. Beach , et al. 2008. “Dissemination of Couples' Interventions Among African American Populations: Experiences From Prosaam.” Family Relations 53: 504–512.

[jmft70008-bib-0031] Kaslow, N. J. , C. Clarke , and J. N. Hampton‐Anderson . 2024. “Culturally Humble and Anti‐Racist Couple and Family Interventions for African Americans.” Family Process 63: 512–526. 10.1111/famp.12938. 37712380

[jmft70008-bib-0032] Kelly, S. , G. Jérémie‐Brink , A. L. Chambers , and M. A. Smith‐Bynum . 2020. “The Black Lives Matter Movement: A Call to Action for Couple and Family Therapists.” Family Process 59, no. 4: 1374–1388. 10.1111/famp.12614. 33217004

[jmft70008-bib-0033] Kenny, D. A. , D. A. Kashy , and W. L. Cook . 2006. Dyadic Data Analysis. Guilford Press.

[jmft70008-bib-0034] Kline, R. B. 2011. Principles and Practice of Structural Equation Modeling. Guilford Press.

[jmft70008-bib-0035] Kogan, S. M. , T. Yu , and G. L. Brown . 2016. “Romantic Relationship Commitment Behavior Among Emerging Adult African American Men.” Journal of Marriage and Family 78, no. 4: 996–1012. 10.1111/jomf.12293. 28989183 PMC5627621

[jmft70008-bib-0036] Van Lange, P. A. 2012. “A History of Interdependence: Theory and Research.” In Handbook of the History of Social Psychology, 341–361. Psychology Press.

[jmft70008-bib-0037] Lavner, J. A. , A. W. Barton , C. M. Bryant , and S. R. H. Beach . 2018. “Racial Discrimination and Relationship Functioning Among African American Couples.” Journal of Family Psychology 32, no. 5: 686–691. 10.1037/fam0000415. 29781635 PMC6072617

[jmft70008-bib-0039] Lemon, R. L. , and C. A. Waehler . 1996. “A Test of Stability and Construct Validity of the Black Racial Identity Scale, Form B (RIAS‐B) and the White Racial Identity Attitude Scale (WRIAS).” Measurement and Evaluation in Counseling and Development 29, no. 2: 77–85. https://psycnet.apa.org/record/1996-05286-002.

[jmft70008-bib-0040] Lind Seal, K. , W. J. Doherty , and S. M. Harris . 2016. “Confiding About Problems in Marriage and Long‐Term Committed Relationships: A National Study.” Journal of Marital and Family Therapy 42, no. 3: 438–450. 10.1111/jmft.12134. 26201911

[jmft70008-bib-0041] McElroy‐Heltzel, S. E. , T. R. Jordan , T. G. Futris , A. W. Barton , A. K. Landor , and K. J. Sheats . 2018. “Sources of Socialization for Interpersonal Trust: An Exploration of Low‐Income Black Adolescents' Experiences.” Journal of Youth Studies 22, no. 1: 124–137. 10.1080/13676261.2018.14920994.

[jmft70008-bib-0042] McNeil Smith, S. , L. D. Williamson , H. Branch , and F. D. Fincham . 2020. “Racial Discrimination, Racism‐Specific Support, and Self‐Reported Health Among African American Couples.” Journal of Social and Personal Relationships 37, no. 3: 779–799. 10.1177/0265407519878519.

[jmft70008-bib-0043] Moore, T. J. , C. Chaney , and A. Skipper . 2021. “‘Put God above All [And He] Will Glorify Your Marriage.’ Relational Spirituality in Black Couples.” Marriage & Family Review 57, no. 8: 673–699. 10.1080/01494929.2021.1887048.

[jmft70008-bib-0044] Murry, V. M. , P. A. Brown , G. H. Brody , C. E. Cutrona , and R. L. Simons . 2001. “Racial Discrimination as a Moderator of the Links Among Stress, Maternal Psychological Functioning, and Family Relationships.” Journal of Marriage and Family 63, no. 4: 915–926. 10.1111/j.1741-3737.2001.00915.x.

[jmft70008-bib-0045] Mussa, K. S. , Z. Dini , and C. M. Bryant . 2023. “Relational Help‐Seeking Among Newlywed African American Couples.” Journal of African American Studies 27, no. 3: 268–282. 10.1007/s12111-023-09632-7.

[jmft70008-bib-0046] Nguyen, A. W. , L. M. Chatters , and R. J. Taylor . 2016. “African American Extended Family and Church‐Based Social Network Typologies.” Family Relations 65, no. 5: 701–715. 10.1111/fare.12218. 28479650 PMC5417543

[jmft70008-bib-1048] Nightingale, M. , C. I. Awosan , and K. Stavrianopoulos . 2019. “Emotionally Focused Therapy: A Culturally Sensitive Approach for African American Heterosexual Couples.” Journal of Family Psychotherapy 30, no. 3: 221–244.

[jmft70008-bib-0048] Parnell, K. J. , and J. H. Hammer . 2018. “Deciding on Couple Therapy: The Role of Masculinity in Relationship Help‐Seeking.” Psychology of Men & Masculinity 19, no. 2: 212–222. 10.1037/men0000098.

[jmft70008-bib-0049] Pew Research Center . 2016. 2016 Racial Attitudes in America Survey. https://www.pewresearch.org/social-trends/dataset/2016-racial-attitudes-in-america-survey/.

[jmft70008-bib-0050] Phillips, T. M. , J. D. Wilmoth , and L. D. Marks . 2012. “Challenges and Conflicts Strengths and Supports: A Study of Enduring African American Marriages.” Journal of Black Studies 43, no. 8: 936–952. 10.1177/0021934712463237.

[jmft70008-bib-0051] Phinney, J. S. 1990. “Ethnic Identity in Adolescents and Adults: Review of Research.” Psychological Bulletin 108, no. 3: 499–514. 10.1037/0033-2909.108.3.499. 2270238

[jmft70008-bib-0052] Powell, W. , L. B. Adams , Y. Cole‐Lewis , A. Agyemang , and R. D. Upton . 2016. “Masculinity and Race‐Related Factors as Barriers to Health Help‐Seeking Among African American Men.” Behavioral Medicine 42, no. 3: 150–163. 10.1080/08964289.2016.1165174. 27337619 PMC4979354

[jmft70008-bib-0053] Preacher, K. J. , and A. F. Hayes . 2008. “Asymptotic and Resampling Strategies for Assessing and Comparing Indirect Effects in Multiple Mediator Models.” Behavior Research Methods 40, no. 3: 879–891. 10.3758/brm.40.3.879. 18697684

[jmft70008-bib-0054] Richman, L. S. , L. P. Kohn‐Wood , and D. R. Williams . 2007. “The Role of Discrimination and Racial Identity for Mental Health Service Utilization.” Journal of Social and Clinical Psychology 26, no. 8: 960–981. 10.1521/jscp.2007.26.8.960.

[jmft70008-bib-0055] Rusbult, C. E. , and P. A. M. Van Lange . 2003. “Interdependence, Interaction, and Relationships.” Annual Review of Psychology 54: 351–375. 10.1146/annurev.psych.54.101601.145059. 12415073

[jmft70008-bib-0056] Settles, I. H. , C. D. Navarrete , S. J. Pagano , C. M. Abdou , and J. Sidanius . 2010. “Racial Identity and Depression Among African American Women.” Cultural Diversity & Ethnic Minority Psychology 16, no. 2: 248–255. 10.1037/a0016442. 20438163

[jmft70008-bib-0057] Skott‐Myhre, H. , and K. Skott‐Myhre . 2022. “The Psychological Colonization of Black Masculinity: Decolonizing Mainstream Psychology for White Allies Working in Mental Health With Black Men.” In Black Men's Health: A Strengths‐Based Approach Through a Social Justice Lens for Helping Professions, edited by Y. D. Dyson , V. Robinson‐Dooley , and J. Watson , 57–67. Springer International Publishing.

[jmft70008-bib-0059] Townes, D. L. , S. Chavez‐Korell , and N. J. Cunningham . 2009. “Reexamining the Relationships Between Racial Identity, Cultural Mistrust, Help‐Seeking Attitudes, and Preference for a Black Counselor.” Journal of Counseling Psychology 56, no. 2: 330–336. 10.1037/a0015449.

[jmft70008-bib-0060] Ulloa, E. C. , J. F. Hammett , N. A. Meda , and S. J. Rubalcaba . 2017. “Empathy and Romantic Relationship Quality Among Cohabitating Couples: An Actor–Partner Interdependence Model.” Family Journal 25, no. 3: 208–214. 10.1177/1066480717710644. PMC10950301 38505465

[jmft70008-bib-0062] Vaterlaus, J. M. , L. Skogrand , and C. Chaney . 2015. “Help‐Seeking for Marital Problems: Perceptions of Individuals in Strong African American Marriages.” Contemporary Family Therapy 37, no. 1: 22–32. 10.1007/s10591-014-9324-4.

[jmft70008-bib-0063] Vowels, L. M. , and K. P. Mark . 2020. “Relationship and Sexual Satisfaction: A Longitudinal Actor–Partner Interdependence Model Approach.” Sexual and Relationship Therapy 35, no. 1: 46–59. 10.1080/14681994.2018.1441991.

[jmft70008-bib-0064] Wallace, B. C. , and M. G. Constantine . 2005. “Africentric Cultural Values, Psychological Help‐Seeking Attitudes, and Self‐Concealment in African American College Students.” Journal of Black Psychology 31, no. 4: 369–385. 10.1177/0095798405281025.

[jmft70008-bib-0065] Williams, D. R. , and S. A. Mohammed . 2009. “Discrimination and Racial Disparities in Health: Evidence and Needed Research.” Journal of Behavioral Medicine 32: 20–47. 10.1007/s10865-008-9185-0. 19030981 PMC2821669

[jmft70008-bib-0066] Wilmoth, J. D. , and A. D. Blaney . 2016. “African American Clergy Involvement in Marriage Preparation.” Journal of Family Issues 37, no. 6: 855–876. 10.1177/0192513X14525619.

[jmft70008-bib-0067] Woods‐Giscombé, C. L. 2010. “Superwoman Schema: African American Women's Views on Stress, Strength, and Health.” Qualitative Health Research 20, no. 5: 668–683. 10.1177/1049732310361892. 20154298 PMC3072704

[jmft70008-bib-0068] Woodward, A. T. , L. M. Chatters , R. J. Taylor , H. W. Neighbors , and J. S. Jackson . 2010. “Differences in Professional and Informal Help Seeking Among Older African Americans, Black Caribbeans, and Non‐Hispanic Whites.” Journal of the Society for Social Work and Research 1, no. 3: 124–139. 10.5243/jsswr.2010.10. 21666782 PMC3111220

[jmft70008-bib-0069] Yap, S. C. Y. , I. H. Settles , and J. S. Pratt‐Hyatt . 2011. “Mediators of the Relationship Between Racial Identity and Life Satisfaction in a Community Sample of African American Women and Men.” Cultural Diversity & Ethnic Minority Psychology 17, no. 1: 89–97. 10.1037/a0022535. 21341901 PMC5551679

[jmft70008-bib-0070] Yeager, C. , and W. J. Doherty . 2022. “African American Marital Confiding Relationships: A National Survey and a Test of an Educational Intervention.” Journal of Marital and Family Therapy 48, no. 2: 411–426. 10.1111/jmft.12506. 33864390

