# Supplementary material for: Identifying Informal Help‐Seeking Patterns in African American Couples
Source: J Marital Fam Ther. 2025 Mar 11;51(2):e70008. doi: 10.1111/jmft.70008 (PMC11995843; doi:10.1111/jmft.70008)
Supplement: Supplementary file 1 — Supporting information. [file JMFT-51-0-s001.docx]

**Supplemental Tables**

**Table 3**

*African American Help-seeking Predictors and Covariates: Descriptive Statistics (N = 350)*

| Variables | *M* | *SD* | Range | α |
| --- | --- | --- | --- | --- |
| Marital Dissatisfaction (W) | 1.31 | .69 | 1 − 5 |  |
| Marital Dissatisfaction (H) | 1.22 | .56 | 1 − 5 |  |
| Income (W) | 7.03 | .16 | 1 − 14 |  |
| Income (H) | 8.29 | .16 | 1 − 14 |  |
| Education (W) | 4.30 | 1.59 | 1 − 7 |  |
| Education (H) | 4.95 | 1.60 | 2 − 7 |  |
| Global Health (W) | 5.70 | 2.04 | 3 − 13 | .96 |
| Global Health (H) | 5.62 | 2.15 | 3 − 15 | .65 |
| Subjective Religiosity (W) | 2.74 | .74 | 1 − 4 |  |
| Subjective Religiosity (H) | 2.64 | .75 | 1 − 4 |  |
| Organizational Religiosity (W) | 7.30 | 2.74 | 3 − 12 | .73 |
| Organizational Religiosity (H) | 6.45 | 2.76 | 3 − 12 | .74 |
| Discrimination (W) | 14.71 | 4.27 | 10 − 36 | .81 |
| Discrimination (H) | 15.12 | 4.92 | 10 − 35 | .83 |
| Internalized Racial ID (W) | 28.09 | 3.50 | 15 − 35 | .65 |
| Internalized Racial ID (H) | 28.43 | 3.54 | 11 − 35 | .67 |
| Pre-Encounter Racial ID (W) | 13.17 | 2.86 | 7 − 22 | .52 |
| Pre-Encounter Racial ID (H) | 13.46 | 3.09 | 7 − 26 | .53 |
| Attitudes toward help-seeking from religious officials (W) | 6.25 | 2.27 | 2 − 10 | .77 |
| Attitudes toward help-seeking from religious officials (H) | 6.08 | 2.20 | 2 − 10 | .81 |

*Note*: Due to the use of only participants who completed all three waves the ranges for husbands and wives are slightly different.

**Table 4**

*Associations between Predictor and Outcome Variables: Correlations and Descriptive Statistics (N = 295 to 350, depending on the wave)*

| Variables | 1 | 2 | 3 | 4 | 5 | 6 | 7 | 8 | 9 | 10 | 11 | 12…. |
| --- | --- | --- | --- | --- | --- | --- | --- | --- | --- | --- | --- | --- |
| 1. Discrimination, Wives T1 | - |  |  |  |  |  |  |  |  |  |  |  |
| 1. Discrimination, Husbands T1 | -.03 | - |  |  |  |  |  |  |  |  |  |  |
| 1. Attitudes toward help-seeking from religious officials, Wives T2 | .04 | -.04 | - |  |  |  |  |  |  |  |  |  |
| 1. Attitudes toward help-seeking from religious officials, Husbands T2 | -.04 | -.01 | .11* | - |  |  |  |  |  |  |  |  |
| 1. Organizational religiosity, Wives T1 | -.02 | -.04 | .22** | .22** | - |  |  |  |  |  |  |  |
| 1. Subjective religiosity item, Wives T1 | -.03 | .03 | -.14* | -.14* | .59** | - |  |  |  |  |  |  |
| 1. Organizational religiosity, Husbands T1 | .003 | .01 | .18** | .31** | .53** | -.31** | - |  |  |  |  |  |
| 1. Subjective religiosity item, Husbands T1 | -.06 | -.10 | .05 | -.22** | .21** | .25** | -.37** | - |  |  |  |  |
| 1. Internalization racial identity, Wives T1 | .04 | .03 | .01 | -.06 | -.01 | .02 | -.05 | .08 | - |  |  |  |
| 1. Pre-Encounter racial identity, Wives T1 | -.092 | -.11 | -.03 | -.04 | -.07 | .11 | .06 | -.01 | -.22** | - |  |  |
| 1. Internalization racial identity, Husbands T1 | -.03 | .18** | -.03 | -.06 | .06 | .02 | -.05 | .07 | .02 | .003 | - |  |
| 1. Pre-Encounter racial identity, Husbands T1 | .01 | -.03 | -.11 | -.01 | -.10** | -.02 | -.02 | -.02 | .02 | .09 | -.16** | - |

*See Table 5 for other correlations.*

**Table 5**

*Continued Correlations and Descriptive Statistics (N = 223 to 350 depending on the missing data for measure/item)*

| Variables Continued | 1 | 2 | 3 | 4 | 5 | 6 | 7 | 8 | 9 | 10 | 11 | 12 | 13 | 14 | 15 | 16 | 17 | 18 |
| --- | --- | --- | --- | --- | --- | --- | --- | --- | --- | --- | --- | --- | --- | --- | --- | --- | --- | --- |
| 1. Religious based help-seeking, Husbands T3 | -.07 | -.11 | .12* | .24** | .22** | -.19** | .12 | -.10 | -.004 | -.11 | .07 | -.08 | - |  |  |  |  |  |
| 1. Informal help-seeking, Husbands T3 | -.01 | -.08 | .08 | .13* | .02 | -.08 | 0.03 | -.04 | -.05 | -.07 | -.12* | .02 | .39** | - |  |  |  |  |
| 1. Formal help-seeking, Husbands T3 | -.05 | -.06 | .03 | .03 | .13 | .02 | .19** | -.06 | -.03 | .02 | -.05 | .10 | -.005 | .05 | - |  |  |  |
| 1. Religious based help-seeking, Wives T3 | .06 | .14* | -.03 | .16* | .25** | -.15** | .15* | -.05 | .01 | -.08 | .07 | .43** | .43** | .05 | -.01 | - |  |  |
| 1. Informal help-seeking, Wives T3 | .22** | -.05 | -.02 | .04 | -.05 | .02 | .03 | -.002 | .06 | -.07 | .03 | .003 | -.03 | -.001 | -.006 | .13* | - |  |
| 1. Formal help-seeking, Wives T3 | .04 | .02 | .04 | .07 | .03 | .02 | .09 | .001 | .02 | -.04 | .07 | -.03 | .08 | .09 | .43** | .07 | .15* | - |
